# Variations in Soil Bacterial Community Diversity and Structures Among Different Revegetation Types in the Baishilazi Nature Reserve

**DOI:** 10.3389/fmicb.2018.02874

**Published:** 2018-11-27

**Authors:** Jiaojiao Deng, You Yin, Wenxu Zhu, Yongbin Zhou

**Affiliations:** ^1^College of Land and Environment, Shenyang Agriculture University, Shenyang, China; ^2^College of Forestry, Shenyang Agriculture University, Shenyang, China; ^3^Research Station of Liaohe-River Plain Forest Ecosystem, Chinese Forest Ecosystem Research Network (CFERN), Shenyang Agricultural University, Shenyang, China

**Keywords:** the Baishilazi Nature Reserve, secondary forests, plantation forest, soil bacterial community, soil characteristics

## Abstract

We compared patterns of soil bacterial community diversity and structure in six secondary forests (JM, *Juglans mandshurica*; QM, *Quercus mongolica*; MB, mixed Broadleaf forest; BE, *Betula ermanii*; CB, conifer-broadleaf forest; PT, *Pinus tabuliformis*) and two plantation forests (LG, Larix gmelinii; PK, *Pinus koraiensis*) of the Baishilazi Nature Reserve, China, based on the 16S rRNA high-throughput Illumina sequencing data. The correlations between the bacterial community and soil environmental factors were also examined. The results showed that the broadleaf forests (JM, QM, MB) had higher levels of total C (TC), total N (TN), available N (AN), and available K (AK) compared to the coniferous forests (PT, LG, PK) and conifer-broadleaf forest (CB). Different revegetation pathways had different effects on the soil bacterial community diversity and structure. For the α-diversity, the highest Shannon index and Simpson index were found in JM. The Simpson index was significantly positively correlated with the available P (AP) (*P* < 0.05), and the Shannon index was significantly positively correlated with AK (*P* < 0.05). Compared with others, the increased ACE index and Chao1 index were observed in the CB and MB, and both of these α-diversity were significantly negative with AK (*P* < 0.05). The relative abundances of bacterial phyla and genera differed among different revegetation types. At the phylum level, the dominant phylum groups in all soils were Proteobacteria, Acidobacteria, Actinobacteria, Verrucomicrobia, Chloroflexi, Bacteroidetes, Gemmatimonadetes, and Planctomycetes. Significant differences in relative abundance of bacteria phyla were found for Acidobacteria, Actinobacteria, Chloroflexi, Gemmatimonadetes, and Proteobacteria. Correlation analysis showed that Soil pH, TC, TN, AP, and AK were the main abiotic factors structuring the bacterial communities. As revealed by the clear differentiation of bacterial communities and the clustering in the heatmap and in the PCA plots, broadleaf forests and coniferous forests harbored distinct bacterial communities, indicating a significant impact of the respective reforestation pathway on soil bacterial communities in the Baishilazi Nature Reserve.

## Introduction

Forests have important ecosystem service functions; therefore, it is crucial to maintain the patterns, functions, and processes of the natural ecosystems. The restoration of the degraded forest ecosystem is a valid approach to solve a number of environmental problems and to increase plant biodiversity, consequently improving ecosystem service functions (Chazdon, 2008). Against this background, research on different forest restoration pathways is of great significance, especially in terms of plant community composition and diversity (Xie et al., 2013). Numerous studies have documented the effects of different revegetation pathways on soil physicochemical characteristics (Jin et al., 2016; Zhang et al., 2018), microbial biomass, and enzyme activities (Li et al., 2018), however, studies on the variations in microbial community composition are scarce (Kang et al., 2018), despite the crucial role of microorganisms in biogeochemical cycles (Smith et al., 2015).

Soil microorganisms, especially bacteria, which represent the most abundant group (Roesch et al., 2007), play central roles in ecosystems, including the carbon, nitrogen, and metals cycling as well as the biodegradation or stabilization of environmental contaminants (Green et al., 2008; He et al., 2011). As a consequence, shifts in the soil microbiome can directly affect soil ecosystem functions, especially carbon and nitrogen cycles (López-Lozano et al., 2013). In forest soils, the physicochemical properties of soil can influence the microbial community diversity, structure, and activity (Fierer and Jackson, 2006). Furthermore, vegetation types are regulatory factors that affect the soil physical and chemical properties (Curlevski et al., 2010; Liu et al., 2012; Oh et al., 2012). Tree species that determine leaf litter quality and quantity can significantly alter soil chemical properties, including soil pH, soil moisture, soil texture (Augusto et al., 2000), soil organic matter content (Frouz et al., 2009), soil nutrient availability (Menyailo et al., 2002), and the relative contents and chemical forms of macronutrients (Hagen-Thorn et al., 2004). This leads to quantitative and qualitative variations in soil carbon and nitrogen pools, combined with soil biological properties (Chen et al., 2004; Curlevski et al., 2010), which can influence the abundance of different bacterial groups (Scherer-Lorenzen and Potvin, 2007; Deyn et al., 2008; Orwin et al., 2010). The rates of microbial decomposition processes both in the litter and soil are substantially influenced by the dominant tree vegetation (Šnajdr et al., 2013). However, the extent to which vegetation type shifts the bacterial community diversity and structure, as well as the relationship between soil physical and chemical properties and the bacterial community are still remain poorly understood.

The Baishilazi Nature Reserve in China is located in the mountainous Region of the Eastern Liaoning Province, China. It was established in 1988 and belongs to the Changbai Mountain system. The original vegetation consisted of broadleaf *Pinus koraiensis* forests, which were severely damaged due to the over-exploitation in the past 100 years. At present, the vegetation mainly consists of natural secondary forests and conifer plantations, and this condition represents a unique opportunity to investigate the soil bacterial community under different reforestation pathways at the same climatic conditions. Numerous studies have investigated the changes in soil microbial biomass (Fan and Liu, 2014), and soil organic carbon contents (Qi, 2017) under different revegetation types, although the structure and diversity of the soil bacterial community have been studied scarcely. In this context, we applied pyrosequencing of the V3–V4 16S rRNA gene to explore both diversity and composition of the soil bacterial community in different revegetation types from eight sites in the Baishilazi Nature Reserve in Liaoning Province, China. Given the differences between conifer and broadleaf forests, we presumed that the soil bacterial community diversity and composition would also differ, although the sites have the same soil type and are subjected to the same climatic. We tested the following hypotheses: (1) different revegetation types impact soil characteristics; (2) the soil bacterial community diversity and structure are linked to soil characteristics; (3) the bacterial community structure of broadleaf forests differ from those of conifer-broadleaf forest and coniferous forests.

The overall goal of our study was to determine the effects of different reforestation pathways on soil bacterial communities. Understanding how plant species alter soil bacterial communities and their related course will conduce to extend our understanding of the biogeochemical elemental cycles that are affected by microbial communities in natural or anthropogenic forest ecosystems.

## Materials and Methods

### Experiment Site

The field study was conducted at the Baishilazi Nature Reserve, in the mountainous region of the eastern Liaoning Province, China (40°50′00′′-40°57′12′′N, 124°44′07′′-124°57′30′′E). The total area of the Baishilazi Nature Reserve encompasses 7,407 hm^2^ and belongs to the mountain range of the Changbai Mountain. This area is characterized by a continental monsoon climate, with warm wet summers, long cold winters, and strong diurnal temperature variation. The mean annual temperature is 6.4°C, with a mean annual precipitation of 1,158 mm. The characteristics of the eight stands are listed in Table 1; all forest stands were older than 40 years.

**Table 1:** Sites information.

Samples	Main herb under the forest	Elevation (m)	Forest type
JM	*Acanthopanax senticosus, Padus racemose, Magnolia sieboldii, Pimpinella brachycarpa, Puccinellia tenuiflora*	901.8	Natural secondary forest
QM	*Acer mono, Cerasus tomentosa, Carpinus cordata*	842.3	Natural secondary forest
MB	*Smilacina japonica, Schisandra chinensis, Viola diamantiaca*	763.4	Natural secondary forest
BE	*Carpinus cordata, Acer mono, Maianthemum bifolium, Schisandra chinensis*	902.9	Natural secondary forest
CB	*Schisandra chinensis, Phryma leptostachya* L. subsp. *asiatica*	826.5	Natural secondary forest
PT	*Hemiptelea davidii, Leymus chinensis*	525.6	Natural secondary forest
LG	*Daemonorops margaritae*	552.7	Plantation forest
PK	*Daemonorops margaritae, Pteridium aquilinum*	552.7	Plantation forest

*JM, Juglans mandshurica; QM, Quercus mongolica; MB, mixed broadleaf forest; BE, Betula ermanii; CB, Conifer-broadleaf forest; PT, Pinus tabuliformis; LG, Larix gmelinii; PK, Pinus koraiensis.*

### Soil Sampling

**FIGURE 1:**
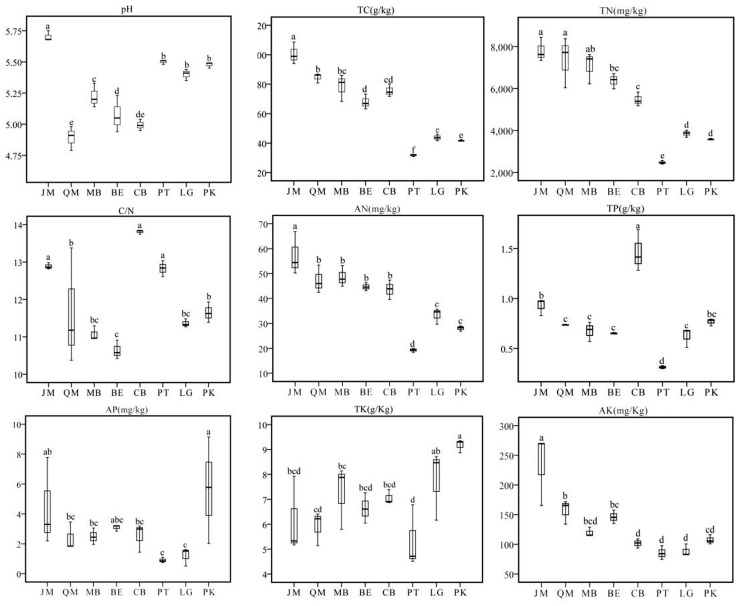
Variations of soil physicochemical properties in JM, *Juglans mandshurica*; QM, *Quercus mongolica*; MB, mixed Broadleaf forest; BE, *Betula ermanii*; CB, Conifer-broadleaf forest; PT, *Pinus tabuliformis*; LG, *Larix gmelinii*; PK, *Pinus koraiensis*.

In July, 2017, we collected soil samples from *Juglans mandshurica* (JM), *Quercus mongolica* (QM), mixed broadleaf forest (MB), *Betula ermanii* (BE), conifer-broadleaf forest (CB), *Larix gmelinii* (LG), *Pinus koraiensis* (PK), and *Pinus tabuliformis* (PT) stands after removal of the litter layer. A total of 24 soil samples (three plots of 20 m × 20 m as three independent replicates) were taken from the A horizon of each stand with the use of a soil auger (8 cm in diameter, 10 cm deep). To ensure the representativeness of soil samples in each stands, the strip sampling method was used. The samples of 15–20 sampling plots within one stand were mixed together to obtain one composite sample per stand. Identically, three real replicate samples stand and placed in cooled boxes. In the laboratory, the samples were sieved through a 2-mm mesh to remove roots and other debris and subsequently divided into two parts. One part was stored at -80°C for DNA extraction, while the other part was air-dried for soil physicochemical analyses. Figure 1 shows the characteristics of the soils from all eight stands.

### Soil DNA Extraction

Soil DNA was extracted from 0.5 g soil, using the Fast DNA SPIN extraction kits (MP Biomedicals, Santa Ana, CA, United States), according to the manufacturer’s instructions. The NanoDrop ND-1000 spectrophotometer (Thermo Fisher Scientific, Waltham, MA, United States) was used to measure the quantity and quality of the extracted DNAs.

### 16S rDNA Sequencing

A quantitative PCR amplification of the bacterial 16S rRNA genes V3–V4 region was performed, using the forward primer 338F (5′-ACTCCTACGGGAGGCAGCA-3′) and the reverse primer 806R (5′-GGACTACHVGGGTWTCTAAT-3′). Sample-specific 7-bp barcodes were incorporated into primers for multiplex sequencing. The PCR amplifications were performed in two steps. During the first step, each of three independent 25 μl mixtures per DNA sample contained 5 μl of Q5 reaction buffer (5×), 5 μl of Q5 High-Fidelity GC buffer (5×), 1 μl (10 μM) of each forward and reverse primer, 2 μl (2.5 mM) of dNTPs, 0.25 μl of Q5 High-Fidelity DNA polymerase (5 U/μl), 2 μl (40–50 ng) of DNA Template, and 8.75 μl of ddH2O. Cycling conditions were 98°C for 5 min (one cycle), 98°C for 15 s, 55°C for 30 s, 72°C for 30 s (25 cycles), followed by 72°C for 5 min. The PCR amplicons were purified with Agencourt AMPure Beads (Beckman Coulter, Indianapolis, IN) and quantified using the PicoGreen dsDNA Assay Kit (Invitrogen, Carlsbad, CA, United States). After the individual quantification step, amplicons were pooled at equal amounts, and pair-end 2 × 300 bp sequencing was performed using the Illumina MiSeq platform with the MiSeq Reagent Kit v3. We have uploaded all raw sequences to the NCBI Sequence Read Archive under submission number SUB4489099 and BioProject number PRJNA489351.

### Statistical Analysis

Sequence data analyses were mainly performed using the software packages QIIME and R (v3.2.0). Operational taxonomic units (OTU)-level alpha diversity indices, such as the Chao1 richness estimator, the abundance-based Coverage Estimator (ACE) metric, the Shannon index, and the Simpson index, were calculated using the OTU table in QIIME. The shared and unique OTUs among samples were used to generate Venn diagrams with the R software package. The linear discriminant analysis (LDA) effect size (LEfSe) algorithm method was used to detect the potential biomarkers based on a normalized relative abundance matrix. The heatmap representation of the relative abundance of bacterial OTUs among samples was built using R. Principal components analysis (PCA) was also conducted based on the genus-level compositional profiles.

Analysis of variance (ANOVA) was performed using SPSS 19.0 software. Soil physicochemical characteristics as well as bacterial total diversity and abundance were compared using LSD tests. Pearson’s correlation analysis was accustomed to estimate the correlations between soil characteristics and bacterial diversity indices. Canonical correspondence analysis (CCA), performed via Canoco 4.5, was used to evaluate the linkages between dominant bacterial groups related to soil environmental factors.

## Results

### Changes in Soil Characteristics

We found significant differences among stands in terms of soil total Carbon (TC) and total Nitrogen (TN) (Figure 1). Interestingly, both TC and TN were highest in JM, with 100.53 and 7.80 g/kg, respectively; while in PT, we measured only 31.76 g/kg and 2.48 g/kg, respectively. Available N (AN) followed the order of JM > MB > QM > BE > CB > LG > PK > PT. In all sites, the soil C/N ratio was below 25:1, with CB showing the highest value. Soil pH value ranged from 4.89 to 5.70; under QM, pH was the lowest with 4.89, followed by CB, while JM had the highest soil pH value. Significant differences were found for available P (AP) and total P (TP), with the highest values under PK and CB (Figure 1).

### Bacterial Community Richness and Diversity Indices of Different Revegetation Types

We obtained a total of 1,448,252 bacterial sequences, which remained after removing low quality sequences and chimeras (on average, 60,343 per sample). In total, 57,139 to 65,460 sequences per sample were obtained and classified into the domain level (Table 2); average sequence length was 330 bp. At the phylum, the remaining sequences were identified for 65,479 OTUs. A maximum of 2,845 OTUs were detected in CB; however, only 2,554 OTUs were obtained in the JM.

**Table 2:** Soil bacterial diversity indexes of different samples.

Sample	No. of sequences	OTUs number (Phylum)	Shannon index	ACE index	Chao1 index	Simpson index
JM	57139 ± 4599b	2554 ± 151b	9.71 ± 0.03a	2635.51 ± 289.86c	2597.17 ± 223.89c	0.9950 ± 0.0002a
QM	59376 ± 5996ab	2710 ± 205ab	9.48 ± 0.07ab	2929.07 ± 553.72bc	2887.56 ± 487.75bc	0.9912 ± 0.0003bc
MB	61986 ± 4055ab	2816 ± 174a	9.32 ± 0.24bcd	3405.73 ± 285.75ab	3368.12 ± 395.94ab	0.9916 ± 0.0014bc
BE	62993 ± 4661ab	2738 ± 48ab	9.20 ± 0.07cd	3236.61 ± 320.61ab	3119.53 ± 410.62abc	0.9906 ± 0.0004bc
CB	65460 ± 1322a	2845 ± 21a	9.28 ± 0.08bcd	3637.79 ± 18.88a	3568.24 ± 149.81a	0.9920 ± 0.0007b
PT	57237 ± 4377b	2754 ± 153ab	9.37 ± 0.05bc	3168.73 ± 499.72abc	3118.16 ± 476.67abc	0.9902 ± 0.0002c
LG	59384 ± 4215ab	2627 ± 61ab	9.10 ± 0.09d	3220.67 ± 108.96ab	3082.82 ± 96.45abc	0.9919 ± 0.0005b
PK	59175 ± 1703ab	2782 ± 217ab	9.46 ± 0.31abc	3365.93 ± 271.01ab	3316.26 ± 318.03ab	0.9939 ± 0.0015a

*Data are means ± standard error (n = 3). JM, Juglans mandshurica; QM, Quercus mongolica; MB, Mixed Broadleaf forest; BE, Betula ermanii; CB, Conifer-broadleaf forest; PT, Pinus tabuliformis; LG, Larix gmelinii; PK, Pinus koraiensis. Different letters in the same line (a, b) indicate a significant difference at P < 0.01.*

Venn diagrams were used to compare the bacterial communities under JM, QM, LG, CB, and PT, based on shared and unique OTUs among the samples. The number of shared OTUs among JM, QM, LG, CB, and PT was 1,187. A total of 392 unique OTUs were found in CB, while 344 unique OTUs were found in LG, 312 in PT, 199 in QM, and 407 in JM. In terms of shared OTUs, 290 were observed between LG and PT, and 502 between QM and JM. Under QM, the lowest number of unique OTUs was found (Supplementary Figure S1), while we detected most unique OTUs under JM.

The different soils showed high species richness, albeit with large variations. The highest species diversity (Shannon and Simpson) was found in JM, with 9.71 and 0.9950, respectively, which also showed the lowest values in ACE and Chao1 index, with 2635.51 and 2597.17, respectively (Table 2). The Shannon index value was lowest under the LG, with 9.10, followed by BE (9.20). However, the Simpson index varied considerably and was lowest under PT (0.9902), followed by BE (0.9906). Highest ACE index and Chao1 index value were observed for MB with 3,405.73 and 3,368.12, respectively, and for CB with 3,637.79 and 3,568.24, respectively, most likely because of the high amounts of leaf litter and roots in these sites. In addition, Person’s rank correlation coefficients showed that the Simpson values were significantly positively correlated with the AP (*P* < 0.05). While, the Shannon value was significantly positively correlated with AK (*P* < 0.05). Values of ACE and Chao1 were significantly negatively correlated with AK (*P* < 0.05) (Table 3).

**Table 3:** Person’s rank correlation coefficients between soil bacterial diversity indices and measured soil characteristics.

	pH	TC	TN	C/N	AN	TP	AP	TK	AK
Shannon	0.405	0.499	0.396	0.365	0.356	0.119	0.572	-0.244	**0.739^∗^**
ACE	-0.414	-0.411	-0.404	0.022	-0.362	0.298	-0.141	0.493	-**0.790^∗^**
Chao1	-0.406	-0.374	-0.377	0.069	-0.346	0.316	-0.100	0.475	-**0.764^∗^**
Simpson	0.576	0.361	0.243	0.255	0.360	0.409	**0.776^∗^**	0.419	0.561

*^∗^Correlation is significant at the 0.05 level (two-tailed). TC, Total Carbon; TN, Total Nitrogen; C/N, oxC-N ration; AN, Available Nitrogen; TP, Total Phosphorus; AP, Available Phosphorus; TK, Total Kalium; AK, Available Kalium.*

The total number of sequences for each sample in the OTU abundance matrix was randomly sampled at different depths. The rarefaction curve was drawn with the number of sequences extracted at each depth and the corresponding OTU numbers. At a genetic distance of 3%, the rarefaction curve flattened with increasing numbers of measured sequences, indicating that the majority of the sample information was obtained (Supplementary Figure S2), adequately reflecting the microbial communities in the soil.

### Bacterial Community Distribution and Composition in Soils

The relative abundances of bacterial phyla and genera differed among different reforestation pathways (Table 4 and Supplementary Figure S3, S4). Across all samples, we detected 34 bacterial phyla and 1 archaeal phyla, of which 10 phyla had an average abundance greater than 1% (Supplementary Figure S3). The dominant phyla were Proteobacteria (39.98–46.77%), Acidobacteria (15.19–27.32%), Actinobacteria (7.03–16.61%), Verrucomicrobia (4.18–7.97%), Chloroflexi (3.03–7.36%), Bacteroidetes (1.82–4.64%), Gemmatimonadetes (1.93–3.15%), Planctomycetes (1.96–2.48%), Firmicutes (0.21–8.50%), and Nitrospiraea (0.35–3.33%) (Table 4), accounting for 97.12–99.07% of the bacterial sequences from each stands. In addition, Saccharibacteria, Cyanobacteria, Latescibacteria, Elusimicrobia, Armatimonadetes, Chlamydiae, and Parcubacteria were present in most soils, albeit at relatively low abundances (below 1%), and all of the other rarer phyla were identified. Among them, Proteobacteria were the absolute dominant species, followed by Acidobacteria and Actinobacteria (Table 4 and Supplementary Figure S3). The relative abundance of Proteobacteria was highest in PT (46.77%) and lowest in PK (39.98%). The relative abundances of Verrucomicrobia and Planctomycetes in all samples did not significantly different. Actinobacteria were less abundant in coniferous forests [PK (8.26%), LG (7.02%), and PT (8.48%)] than in broadleaf forests [MB (16.40%), JM (15.88%), QM (14.82%), and BE (16.61%)] and conifer-broadleaf forest (14.93%). Firmicutes dominated in PK and were rare in the other stands. While JM had the highest relative abundances of Chloroflexi (7.36%), Gemmatimonadetes (3.15%), and Nitrospirae (3.33%), but the lowest abundances of Acidobacteria (15.19%) and Firmicutes (0.21%) (Table 4 and Supplementary Figure S3).

**Table 4:** Relative abundance of the most abundant bacterial phyla (>1%) present in the soil samples.

Samples	Proteobacteria (%)	Acidobacteria (%)	Actinobacteria (%)	Verrucomicrobia (%)	Chloroflexi (%)
JM	41.31 ± 0.59cd	15.19 ± 1.27e	15.87 ± 2.85a	6.94 ± 1.97a	7.36 ± 0.11a
QM	45.11 ± 1.19ab	19.33 ± 0.96cd	14.80 ± 2.94a	6.38 ± 1.80a	4.16 ± 0.12cd
MB	44.99 ± 0.31ab	19.01 ± 1.02cd	16.40 ± 3.17a	5.72 ± 4.92a	4.92 ± 1.58bcd
BE	44.08 ± 0.15ab	17.73 ± 1.31de	16.61 ± 4.72a	7.97 ± 4.13a	4.75 ± 0.85bcd
CB	44.12 ± 0.73ab	23.60 ± 0.68b	14.93 ± 1.62a	5.59 ± 0.57a	3.03 ± 0.35d
PT	46.77 ± 1.02a	21.52 ± 2.47bc	8.48 ± 2.16b	5.25 ± 1.46a	6.50 ± 0.53ab
LG	42.97 ± 0.82bc	27.32 ± 0.75a	7.03 ± 3.15b	7.22 ± 2.59a	5.39 ± 0.24bc
PK	39.98 ± 3.94d	19.13 ± 2.46cd	8.26 ± 2.48b	4.18 ± 1.27a	6.58 ± 2.48ab

**Samples**	**Bacteroidetes (%)**	**Gemmatimonadetes (%)**	**Planctomycetes (%)**	**Firmicutes (%)**	**Nitrospirae (%)**

JM	3.26 ± 0.42ab	3.15 ± 0.14a	1.97 ± 0.34a	0.21 ± 0.03b	3.33 ± 0.29a
QM	2.87 ± 0.36ab	2.83 ± 0.31ab	2.10 ± 0.42a	0.55 ± 0.09b	0.75 ± 0.46de
MB	1.82 ± 1.19b	2.41 ± 1.01abc	1.95 ± 0.71a	0.56 ± 0.13b	1.29 ± 0.53bcd
BE	1.61 ± 0.63b	2.19 ± 0.43bc	2.48 ± 0.03a	0.46 ± 0.15b	1.07 ± 0.22cd
CB	1.89 ± 0.26b	1.93 ± 0.09c	2.34 ± 0.26a	0.88 ± 0.04b	0.35 ± 0.07e
PT	2.58 ± 0.06b	3.12 ± 0.22a	2.29 ± 0.45a	0.52 ± 0.26b	0.94 ± 0.08d
LG	1.97 ± 0.15b	2.22 ± 0.39bc	2.17 ± 0.25a	0.55 ± 0.29b	1.53 ± 0.17bc
PK	4.64 ± 2.86a	2.17 ± 0.30bc	1.96 ± 0.40a	8.50 ± 7.83a	1.70 ± 0.46b

*Values are means ± standard deviation (n = 3). JM, Juglans mandshurica; QM, Quercus mongolica; MB, Mixed Broadleaf forest; BE, Betula ermanii; CB, Conifer-broadleaf forest; PT, Pinus tabuliformis; LG, Larix gmelinii; PK, Pinus koraiensis. Different letters in the same line (a,b) indicate a significant difference at P < 0.01.*

At the genus level, average relative abundances below 1% were grouped into “others,” and 11 groups were obtained. The relative abundances of several genera differed significantly between the different samples (Supplementary Figure S4). The soil was dominated by *Nitrobacter* (6.49%), followed by *Candidatus-Solibacte*r (3.72%), *Acidothermus* (2.86%), *Pseudolabrys* (2.43%), and *Bryobacter* (1.98%). Significant differences in relative abundance were not found for the genera *Variibacter* and *Haliangium* among samples. The relative abundances of *Nitrobacter, Candidatus-Solibacter, Acidothermus, Pseudolabrys, Bryobacter, Rhizomicrobium, H16*, and *Reyranella* showed significant differences between JM and QM. Similarly, the relative abundances of *Nitrobacter, Candidatus-Solibacter, Bryobacter*, and *H16* differed significantly among the JM, PT, and CB. The stand CB had the highest relative abundances of *Candidatus-Solibacter, Acidothermus, Bryobacter*, and *Rhizomicrobium*, but the lowest abundance of *H16*. PT had a significantly higher relative abundance of *Nitrobacter* compared to the other stands. Significant differences in relative abundance between LG and PK were found for the genera *Nitrobacter, Candidatus-Solibacter, Acidothermus, Bryobacter*, and *H16* (Supplementary Figure S4).

**FIGURE 2:**
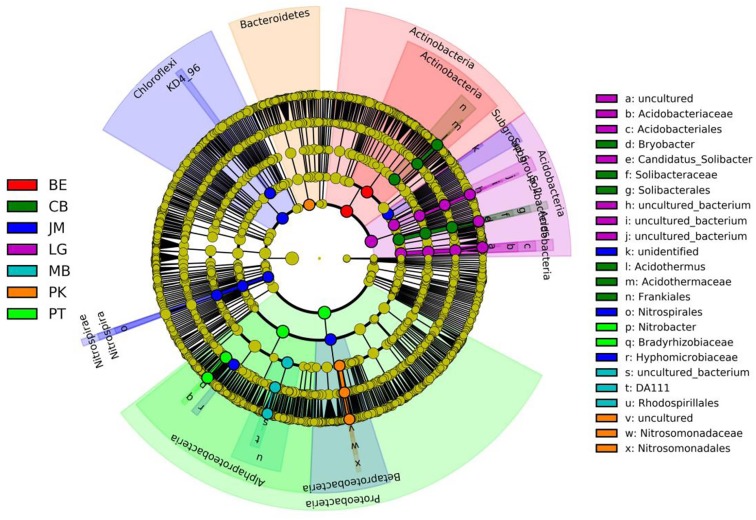
Cladogram of soil bacterial in revegetation types via LEfSe method identifies the significantly different abundant taxa. Circle radiation from inner to outer of evolutionary branch figure represents the classification of the level from phylum doorgenus; every small circle represents the level of a classification in different classification level. The diameter of the circle is proportional to the relative abundance; the principle is that the species without significant differences uniformly color to yellow, and the other species are colored according to the highest abundance of the species. JM, *Juglans mandshurica*; QM, *Quercus mongolica*; MB, mixed broadleaf forest; BE, *Betula ermanii*; CB, Conifer-broadleaf forest; PT, *Pinus tabuliformis*; LG, *Larix gmelinii.*

The LEfSe analysis was documented to determine the classified bacterial taxa with significant abundance differences among the different forest stands. As presented in Figure 2, 39 bacterial taxa were showed significantly different with LDA effect size scores were >4. At the phylum level, the biomarkers were affiliated with Proteobacteria, Acidobacteria, Actinobacteria, Chloroflexi, and Bacteroidetes.

**FIGURE 3:**
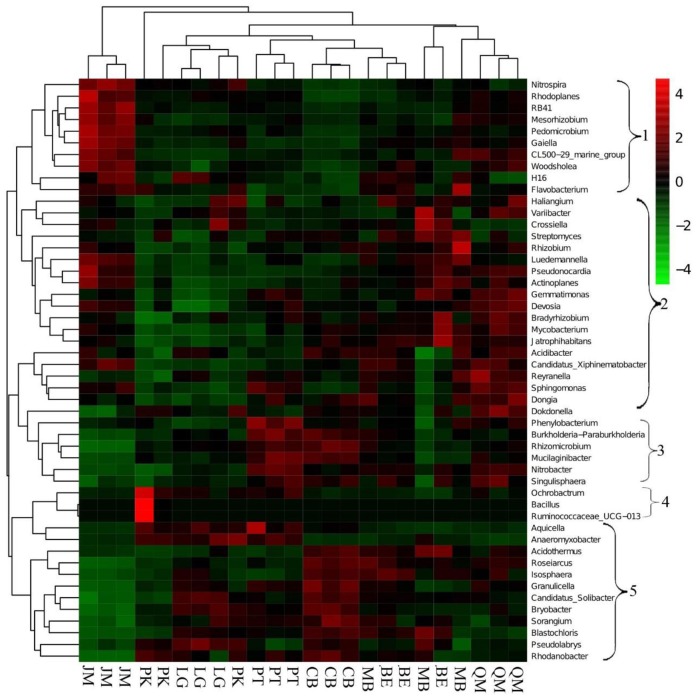
Heatmap and hierarchical cluster analysis based on the relative abundances of the top 50 genera identified in the bacterial communities of the soils. The samples are grouped according to the similarity of each other, and the clustering results are arranged horizontally according to the clustering results. In the figure, red represents the genus with higher abundance in the corresponding sample, and green represents the genus with lower abundance. JM, *Juglans mandshurica*; QM, *Quercus mongolica*; MB, Mixed Broadleaf forest; BE, *Betula ermanii*; CB, Conifer-broadleaf forest; PT, *Pinus tabuliformis*; LG, *Larix gmelinii*; PK, *Pinus koraiensis.*

**FIGURE 4:**
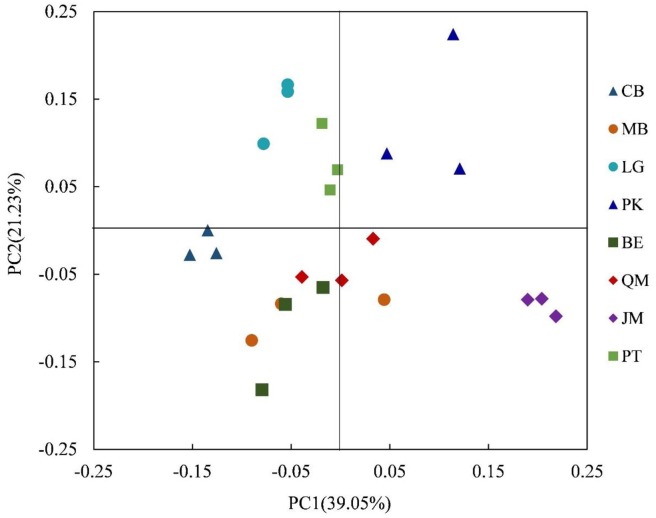
Principal component analysis of the composition of bacterial communities in the soil of forests with different dominant trees. JM, *Juglans mandshurica*; QM, *Quercus mongolica*; MB, Mixed Broadleaf forest; BE, *Betula ermanii*; CB, Conifer-broadleaf forest; PT, *Pinus tabuliformis*; LG, *Larix gmelinii*; PK, *Pinus koraiensis.*

We applied heatmap analysis to intuitively display the differences in relative abundances of the top 50 bacterial genera that appeared in all soil samples (Figure 3), which can reflect the differences in soil bacterial community structure between different vegetation types. The resulting heatmap could be divided into five clusters. Both relative abundance and distribution of soil bacteria in different stands were significantly different. In Cluster 1, higher relative abundances of 10 bacterial genera were found in JM, and significant differences were found for the other seven stands. The 19 bacterial genera with higher relative abundances in Cluster 2 were mainly found in QM, BE, and MB. In Cluster 3, the higher relative abundances of the six bacterial genera were mainly found in the PT and CB, and the three bacterial species with higher relative abundances in Cluster 4 were mainly found in PK and were rare in other stands. The three bacterial genera with higher relative abundances in Cluster 5 were mainly found in LG, CB, and MB. This indicates that the compositions and relative abundance of soil bacteria differed significantly between the stands. To further explore these differences, we applied principal components analysis (PCA) to extract the main components. The results of the PCA showed that soil bacterial community from the coniferous forest stand was separated from those of coniferous-broadleaf forest and the broadleaf forest, especially along PC2 (Figure 4).

### Effect of Tree Species on Soil Properties and Microorganisms

**FIGURE 5:**
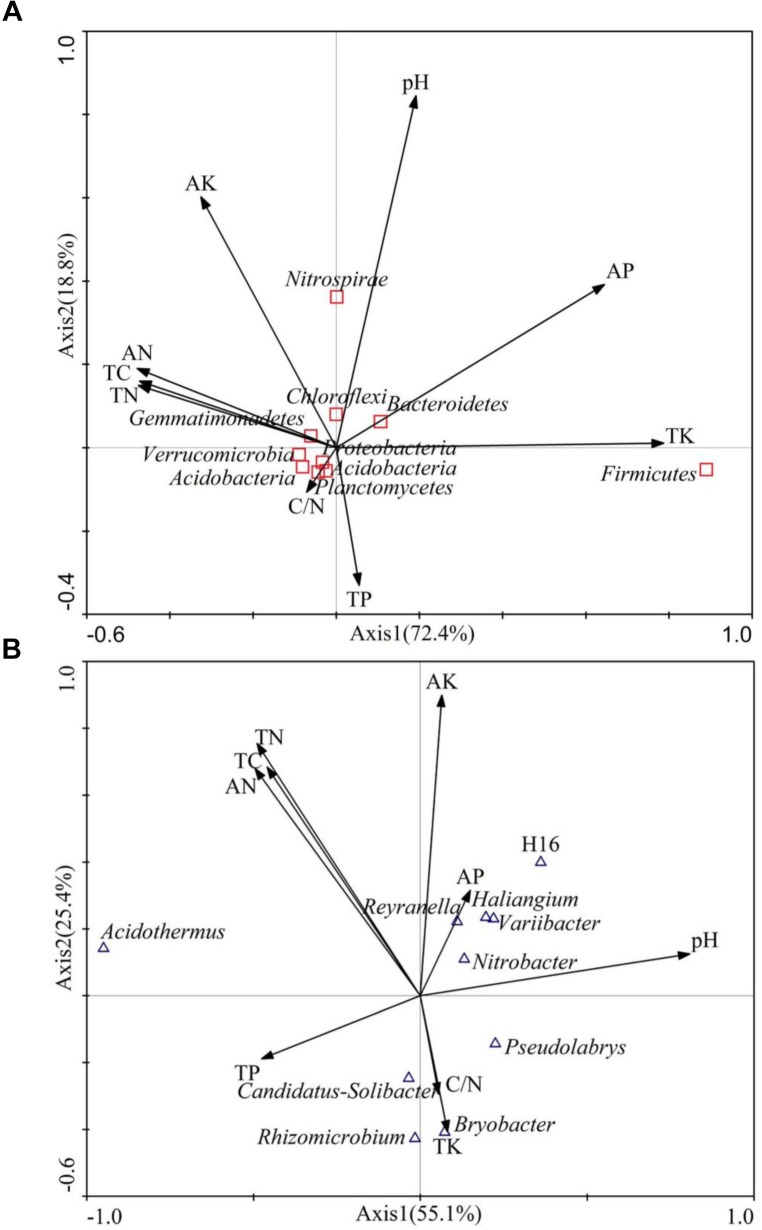
Canonical correspondence analysis (CCA) on soil dominant bacteria phyla **(A)** and soil dominant bacteria genus **(B)** constrained by soil variables.

Canonical correspondence analysis (CCA) was applied to explore the relative abundance of dominant bacteria phyla and genera as a function of soil variables (Figure 5). The first and second axes accounted for 91.2% and 80.5% of the variation, respectively, indicating that soil environmental factors significantly influence the bacterial community structure. At the phylum level (Figure 5A), AP (*r* = 0.6453) and TK (*r* = 0.7877) were significantly associated with axis1, which explained 72.4% of the variation. In contrast, soil pH (*r* = 0.8449) and AK (*r* = 0.6020) were significantly associated with axis 2. At the genus level (Figure 5B), pH (*r* = 0.8079) was associated with axis 1, accounting for 55.1% of the variation, while TC(*r* = 0.6839), TN (*r* = 0.7548), AN (*r* = 0.6798), and AK (*r* = 0.8993) were associated with axis 2. Based on this, soil pH, TC, TN, AN, TK, and AK significantly influenced the bacterial community structure.

We investigated the relationships between the relative abundances of different dominant bacterial phyla and genera and environmental variables (Table 5). In terms of soil chemical properties, AP was negatively correlated with the relative abundances of Proteobacteria, but positively correlated with Bacteroidetes abundance, while Acidobacterias’ relative abundance was negatively correlated with AK (*P* < 0.05). The relative abundance of Actinobacteria was significantly positively correlated with TC, TN, and AN (*P* < 0.01), and Chloroflexi relative abundance was significantly positively correlated with pH (*P* < 0.01). The relative abundances of Firmicutes was positively correlated with TK (*P* < 0.01).

**Table 5:** Person’s rank correlations between the relative abundances of dominant bacteria groups and measured soil characteristics.

Bacteria group	pH	TC	TN	C/N	AN	TP	AP	TK	AK
Phylun	–	–	–	–	–	–	–	–	–
Proteobacteria	-0.456	-0.093	-0.047	0.044	-0.162	-0.316	-**0.837^∗∗^**	-0.706	-0.337
Acidobacteria	-0.169	-0.560	-0.594	0.121	-0.505	0.042	-0.629	0.227	-**0.772^∗^**
Actinobacteria	-0.445	**0.871^∗∗^**	**0.894^∗∗^**	-0.031	**0.860^∗∗^**	0.415	0.177	-0.355	0.614
Verrucomicrobia	-0.168	0.387	0.469	-0.351	0.504	-0.067	-0.271	-0.359	0.426
Chloroflexi	**0.932^∗∗^**	-0.215	-0.234	-0.026	-0.225	-0.509	0.357	0.025	0.322
Bacteroidetes	0.475	-0.150	-0.204	0.124	-0.232	-0.046	**0.716^∗^**	0.377	0.200
Gemmatimonadetes	0.452	0.184	0.144	0.210	0.055	-0.464	-0.129	-0.716^∗^	0.498
Planctomycetes	-0.440	-0.235	-0.230	0.073	-0.217	0.039	-0.461	-0.346	-0.307
Firmicutes	0.235	-0.415	-0.409	-0.108	-0.414	0.029	0.701	**0.771^∗^**	-0.230
Nitrospirae	**0.796^∗^**	0.317	0.271	0.004	0.349	-0.078	0.520	0.075	0.702
Genus	–	–	–	–	–	–	–	–	–
*Nitrobacter*	-0.491	-0.212	-0.150	-0.015	-0.296	-0.406	-**0.754^∗^**	-0.700	-0.340
*Candidatus-Solibacter*	-0.550	-0.336	-0.321	-0.016	-0.256	0.210	-0.626	0.177	-**0.737^∗^**
*Acidothermus*	-**0.809^∗^**	0.406	0.461	-0.110	0.451	0.452	-0.215	-0.099	-0.090
*Pseudolabrys*	-0.183	-0.453	-0.435	-0.175	-0.325	0.223	-0.082	**0.738^∗^**	-0.626
*Bryobacter*	-0.415	-0.530	-0.573	0.199	-0.502	0.245	-0.500	0.264	-**0.835^∗^**
*Variibacter*	-0.423	0.349	0.495	-0.530	0.397	-0.199	-0.254	-0.126	0.205
*Haliangium*	-0.518	0.381	0.559	-0.666	0.465	-0.083	0.165	-0.069	0.400
*Rhizomicrobium*	-0.503	-0.476	-0.539	0.372	-0.541	0.192	-0.531	-0.053	-**0.791^∗^**
*H16*	0.578	0.262	0.295	-0.282	0.400	-0.179	0.077	0.056	0.459
*Reyranella*	-0.650	0.221	0.334	-0.256	0.172	-0.280	-0.607	-0.613	0.002

*TC, Total Carbon; TN, Total Nitrogen; C/N, oxC-N ration; AN, Available Nitrogen; TP, Total Phosphorus; AP, Available Phosphorus; TK, Total Kalium; AK, Available Kalium. ^∗∗^Correlation significant at 0.01 level (two-tailed); ^∗^Correlation significant at 0.05 level (two-tailed).*

At the genus level, *Acidothermus* was significantly negatively correlated with pH, while *Nitrobacter* was significantly negatively correlated with AP. The abundance of *Pseudolabrys* was positively correlated with TK, while *Candidatus-Solibacter, Bryobacter*, and *Rhizomicrobium* were significantly negatively correlated with AK.

## Discussion

### Different Revegetation Types Contribute to Different Soil Physicochemical Characteristics

Reforestation pathways greatly affected the soil physicochemical characteristics through various processes, including changes in moisture and temperature, as well as the production of litter and root exudates. In our study, we observed that the broadleaf forests (JM, QM, MB) had higher levels of TC, TN, AN, and AK compared to the coniferous forests (PT, LG, PK) and the conifer-broadleaf forest (CB) (Figure 1), which is consistent with previous research suggesting that broadleaf-Korean pine mixed forest and secondary poplar-birch forest presented significantly higher OC and TN values than spruce fir and larch forest (Hui et al., 2014). The higher TC and TN values in the three broadleaf forests are most likely a result of constant organic matter inputs, rhizodeposition, and the release and recycling of nutrients (Finér et al., 2007; Wang and Cao, 2011). In complete contrast to the TC and TN pattern, the soil C/N ratios in coniferous forests (PT, LG, and PK) and the conifer-broadleaf forest were significantly higher than those in broadleaf forests (QM, BE, and MB). Previous studies have also reported that coniferous forests tend to be highly nitrogen deficient compared with broadleaf forests (Vitousek and Howarth, 1991; Mcgroddy et al., 2004). The soil C/N ratio directly reflects the soil’s nitrogen mineralization capacity, and low soil C/N levels indicate a higher nitrogen mineralization rate, facilitating N uptake by microbes and plants. In turn, high C/N levels are beneficial for the fixation the of soil organic carbon. Litter C/N ratios of coniferous forests are generally higher than those of broadleaf forests, which may contribute to the higher C/N ratio in coniferous forest soils (Yang and Luo, 2011). Several studies have reported that higher C/N ratios and lower nutrient contents in coniferous forests compared with broadleaf forests (Barbier et al., 2008). In our study area, the Liaodong mountainous area, the soil was relatively acidic, with pH value ranging from 4.89 to 5.70, with the lowest levels under QM (pH = 4.89). These differences in pH may be due to different foliage properties and variations in litter quality (Haghdoost et al., 2011). Soil pH, nutrient contents, and numerous biogeochemical processes in forest ecosystems can be influenced by litter chemistry (Mueller et al., 2012; Fanin and Bertrand, 2016). Compared to other broadleaf forests, litter leaf quality in QM was relatively low, with a low nitrogen content, a high C/N ratio, a higher lignin content, and higher lignin/N. Therefore, the decomposition rate of QM litter and the release rates of plant nutrients decreased gradually.

### Response of Bacterial Community Diversity to Soil Physicochemical Characteristics in Different Reforestation Sites

Previous studies have demonstrated the crucial role of soil characteristics in altering the soil microbial communities during vegetation restoration (Thomson et al., 2015). Soil pH and nutrient contents, especially in terms of C, N, and P availability, are paramount factors (Ramirez et al., 2010; Tan et al., 2013) and significantly affect microbial abundance (Christianl et al., 2008; Paulinem and Davide, 2008; Zhong et al., 2010). When soil pH was below 6.5, the pH was supposed to be the primary factor which effected bacterial community diversity, structure and activity, and higher richness and diversity values were found at a pH close to neutral (Preem et al., 2012; Ramirez et al., 2012; Bergkemper et al., 2016). In contrast, Lauber et al. (2009) has proposed that there is a significantly negative relationship between soil pH and bacterial diversity. However, in my study, soil pH was not significantly correlated with any bacterial diversity index. However, we observed a relatively small pH rangs (4.89 to 5.70), making it different to find any correlation with bacterial diversity. Previous studies have suggested that bacterial alpha diversity is largely affected by SOC and TN (Siles and Margesin, 2016; Ren et al., 2017). However, in our study, TC and TN levels were not significantly correlated with bacterial diversity indexes, which was consistent with the observations obtained by Lin et al. (2014).

In most terrestrial ecosystems, the soil C/N ratio greatly influences soil microbial communities (Fierer and Jackson, 2006) and is negatively correlated with the soil bacterial Shannon index (Zhou et al., 2017). However, in our study, C/N ratio showed no correlation with bacterial diversity indices, most likely because of the relatively small C/N range (10.64–13.81).

However, the Simpson index was significantly positively correlated with the AP, which is in accordance with a previous study suggesting that P increases bacterial diversity in pasture soil (Tan et al., 2013). Similarly, soil P is the second most significant driver of bacterial diversity in soil (Siciliano et al., 2014), although some authors have stated that bacterial diversity was not affected by P levels (Liu et al., 2018). In our study, the Shannon value was significantly positively correlated with AK (*P* < 0.05) (Table 3) and increased ACE index and Chao1 index values were observed in CB and MB, reflecting the large amounts of leaf litter and roots. Both of these α-diversity indicators were significantly negatively correlated with AK (Table 3), which leads us to infer that AP and AK availability may be the limiting factor affecting bacterial diversity in this area.

### Response of the Bacterial Taxonomy to Different Revegetation Types

Similar to bacterial community diversity, the relative abundances of dominant bacterial phyla were influenced by different revegetation types. With regard to bacterial compositions, the different revegetation forest stands harbored distinct bacterial communities. In our study, Proteobacteria, Acidobacteria, and Actinobacteria were the most abundant soil bacterial phyla in the Baishilazi Nature Reserve, which is in agreement with a past study in northern soils (Sun et al., 2014). In the light of bacterial classification, Proteobacteria, Acidobacteria, Actinobacteria, Firmicutes, Gemmatimonadetes, Nitrospirae, and Chloroflexi significantly differed between the different forest stands (Table 4). Acidobacteria was the dominant taxa in LG, while Actinobacteria was the dominant taxa in PK; Proteobacteria dominated the soil under PT. In constrast, Nitrospirae and Chloroflexi were dominant in JM. In contrast to our study, previous studies reported that coniferous forests were dominated by Proteobacteria and Gemmatimonadetes (Hui et al., 2014).

Proteobacteria was the most abundant phylum in our research, which is similar to the result of previous studies (Li et al., 2014a; Miyashita, 2015). However, according to Liu et al. (2013), Actinobacteria play a dominant role in the bacterial community of the mixed forest soil of the Dinghushan Mountain. Generally, members of the Proteobacteria and Acidobacteria are ubiquitous in almost all soil types (Zhang and Xu, 2008). In our study, despite different revegetation types, including broadleaf forests, coniferous forests, and conifer-broadleaf forest, the composition of bacterial groups was similar for all phyla detected. Previous studies have suggested that the Proteobacteria can be used as indicators of the prevalent nutrient status due to their different natures (Hartman et al., 2008). In our study, the relative abundance of Proteobacteria was highest in PT and largely affected by AP, but not by measured C, although a high abundance of Proteobacteria has previously been associated with a high C availability (Fazi et al., 2005; Fierer et al., 2007). This may be related to the composition of Proteobacteria in the different forest stands, and our results indicated that in the study region, soil TC had no significant impact on the bacterial community composition.

Recent studies have shown that Acidobacteria are generally oligotrophic (Kielak et al., 2009; López-Lozano et al., 2013), and Acidobacteria play a key role in biogeochemical nutrient cycling (Tringe et al., 2005). Apparently, the level of nitrogen input is negatively correlated with the relative abundance of Acidobacteria (Fierer et al., 2012), which is line with the results of our study. Acidobacteria belonged to the acidophilic bacteria, and Acidobacteria abundance is significantly higher under acidic conditions (Dion, 2008; Lauber et al., 2009; Bardhan et al., 2012). However, in our study, the relative abundance of the Acidobacteria was not correlated with soil pH (Table 5) but was significantly negatively correlated with AP. Soil properties, especially AP and AK, were significantly correlated with the bacterial, even at the very small geographical scale in our study, and this finding is in line with the previous research (Cai et al., 2018).

In our study, except for the Proteobacteria and Acidobacteria, the significant difference in the relative abundance of Actinobacteria was observed in coniferous forests (PK, LG, and PT) and was lower than the abundance in broadleaved forests (JM, QM, and BE). This finding is in agreement with a previous which reported that Actinobacteria are among the most important organic matter decomposers in soil (Kopecky et al., 2011). In our study, the differences in relative Actinobacteria abundance between the different reforestation pathways were related to changes in TC, TN, and AN (Figure 5 and Table 5), which is in agreement with a previous study stating that Actinobacteria are significantly positively correlated with the soil organic matter accumulation, during secondary forest succession (Guo et al., 2018). In addition, PK showed a higher relative abundance of Firmicutes compared to the other forest stands, confirming the high resistance of these bacteria to unfavorable conditions (Hartmann et al., 2014). Other specific taxa, particularly Chloroflexi and Nitrospirae, were significantly correlated with the soil pH (Table 5).

The clear differentiation of bacterial communities and the clustering in the heatmap (Figure 3) and in PCA (Figure 4) plots, suggests that broadleaf forests and coniferous forests harbored different bacterial communities, indicating that the soil bacterial community composition largely depends on soil physicochemical characteristic. Soil physicochemical variables therefore play the decisive role in altering bacterial communities during vegetation restoration, which is consistent with previous studies (Kuramae et al., 2010; Li et al., 2014b).

## Conclusion

Our results indicate that different revegetation types in the Baishilazi Nature Reserve have different impacts on the soil physicochemical characteristics and the bacterial communities. Broadleaf forests had higher TC, TN, AN, and AK values when compared to coniferous forests. In the study area, AP and AK availability are limiting factors and significantly affect bacterial diversity. Broadleaf forests, conifer-broadleaf forests, and coniferous forests harbored significantly different bacterial communities. Our study contributes to the understanding of the responses of bacterial communities to different reforestation pathways in mountainous regions.

## Author Contributions

All authors commented on the manuscript at all stages. JD and YY conceived and designed the study. JD and WZ contributed materials and analysis tools. JD, YY, WZ, and YZ contributed to the data analysis and paper preparation.

## Conflict of Interest Statement

The authors declare that the research was conducted in the absence of any commercial or financial relationships that could be construed as a potential conflict of interest.
