# Supplementary figures and images for: Variations in Soil Bacterial Community Diversity and Structures Among Different Revegetation Types in the Baishilazi Nature Reserve
Source: Front Microbiol. 2018 Nov 27;9:2874. doi: 10.3389/fmicb.2018.02874 (PMC6277578; doi:10.3389/fmicb.2018.02874)

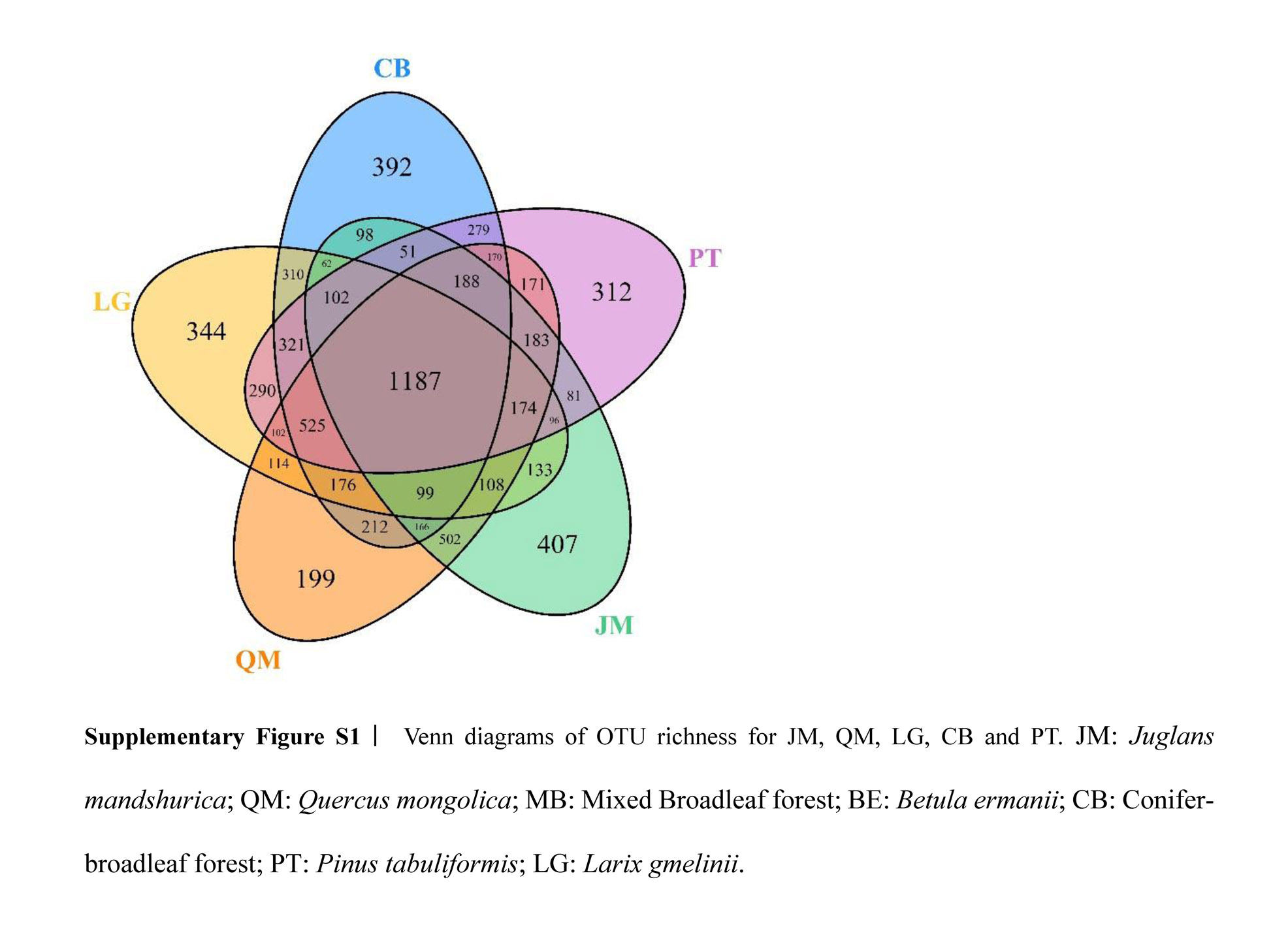

Supplement: Supplementary file 1 [file Image_1.JPEG]

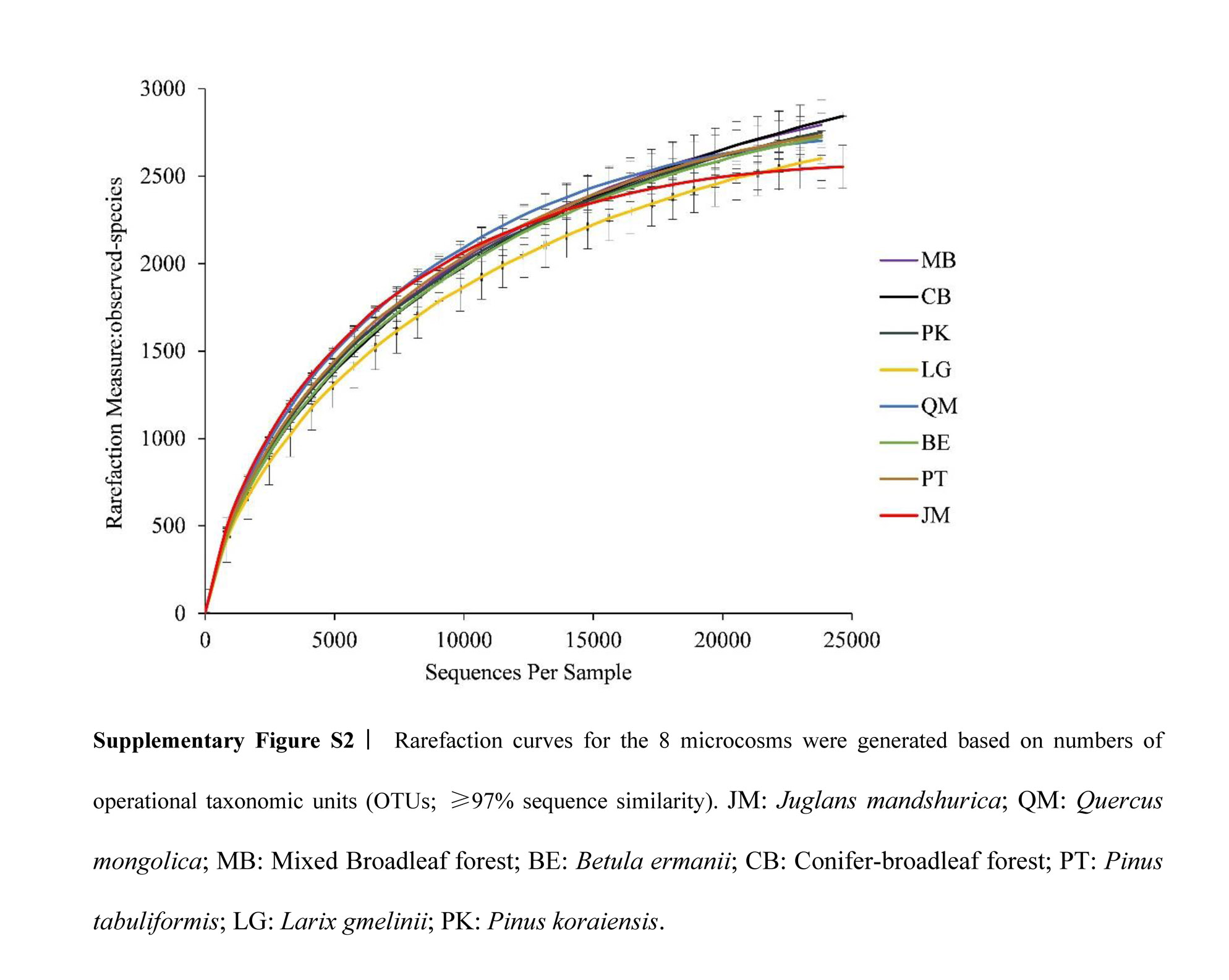

Supplement: Supplementary file 2 [file Image_2.JPEG]

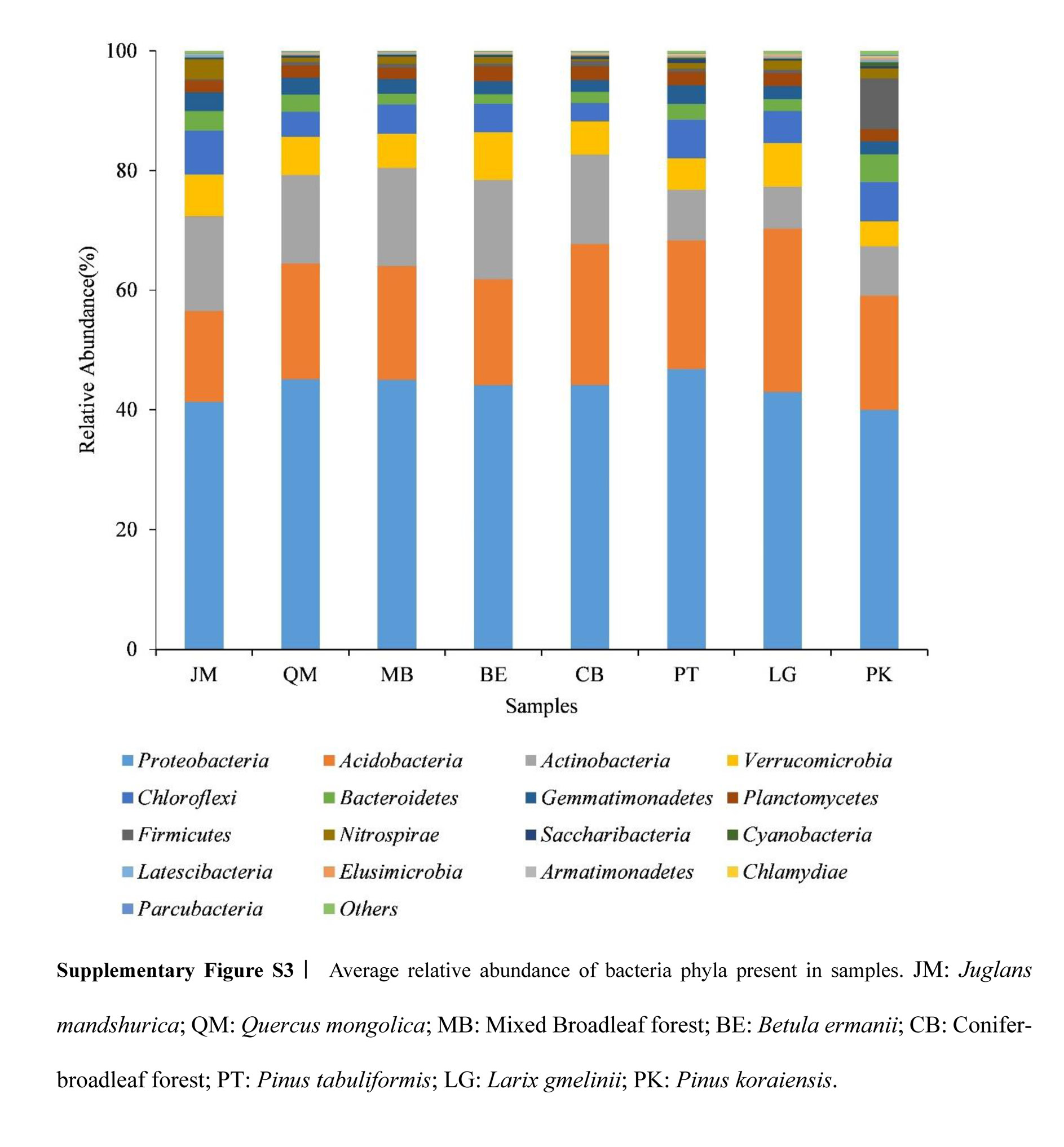

Supplement: Supplementary file 3 [file Image_3.JPEG]

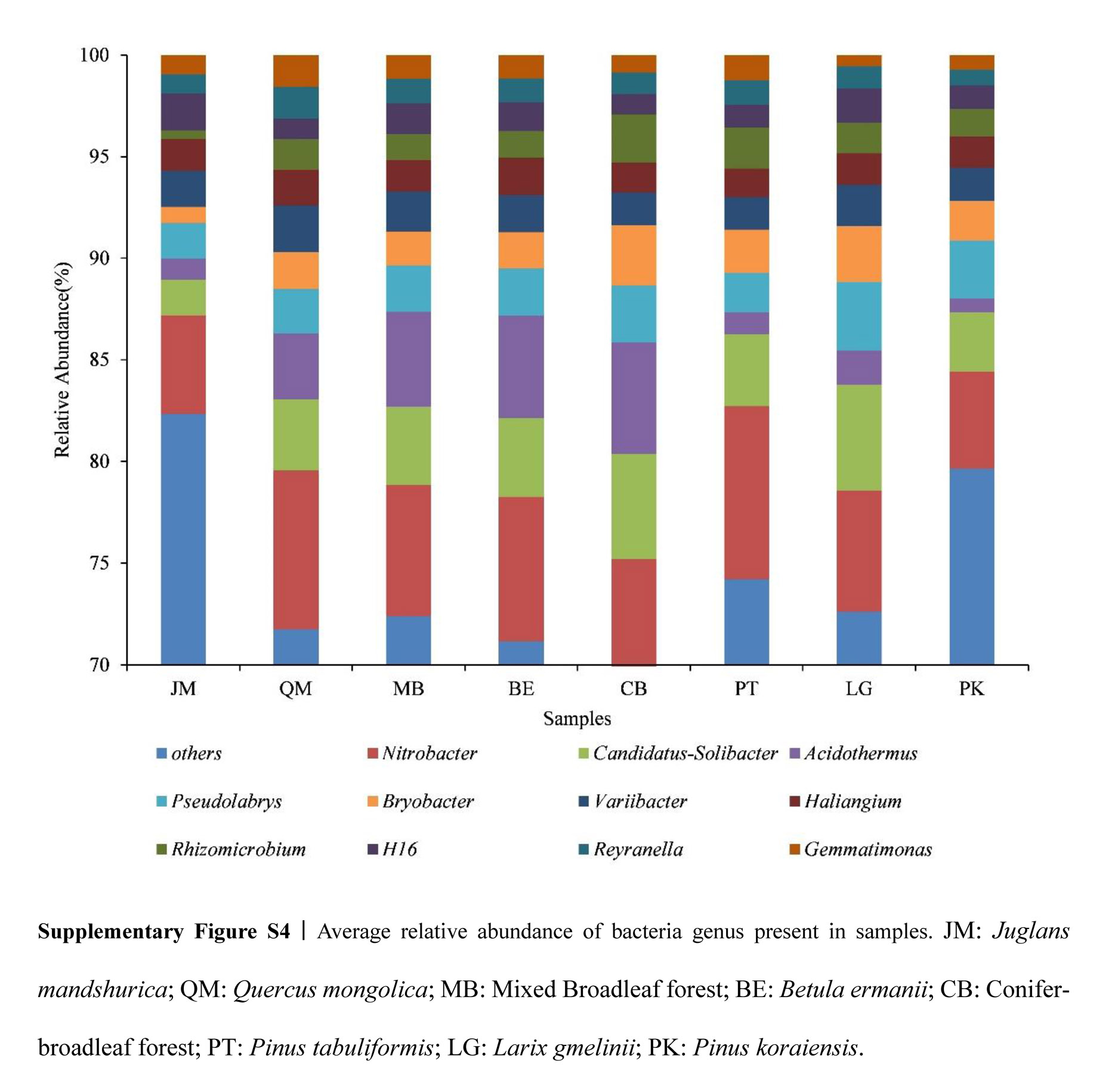

Supplement: Supplementary file 4 [file Image_4.JPEG]
